# The Intersection of IgE Autoantibodies and Eosinophilia in the Pathogenesis of Bullous Pemphigoid

**DOI:** 10.3389/fimmu.2019.02331

**Published:** 2019-10-04

**Authors:** Kelly N. Messingham, Tyler P. Crowe, Janet A. Fairley

**Affiliations:** ^1^Department of Dermatology, The University of Iowa, Iowa City, IA, United States; ^2^Iowa City VA Medical Center, Iowa City, IA, United States

**Keywords:** IgE, eosinophil, Bullous pemphigoid, autoimmunity, autoantibody, blister, skin, collagen XVII

## Abstract

Bullous pemphigoid (BP) is an autoimmune blistering disease characterized by autoantibodies targeting cellular adhesion molecules. While IgE autoantibodies are occasionally reported in other autoimmune blistering diseases, BP is unique in that most BP patients develop an IgE autoantibody response. It is not known why BP patients develop self-reactive IgE and the precise role of IgE in BP pathogenesis is not fully understood. However, clinical evidence suggests an association between elevated IgE antibodies and eosinophilia in BP patients. Since eosinophils are multipotent effector cells, capable cytotoxicity and immune modulation, the putative interaction between IgE and eosinophils is a primary focus in current studies aimed at understanding the key components of disease pathogenesis. In this review, we provide an overview of BP pathogenesis, highlighting clinical and experimental evidence supporting central roles for IgE and eosinophils as independent mediators of disease and via their interaction. Additionally, therapeutics targeting IgE, the Th2 axis, or eosinophils are also discussed.

## Overview of Bullous Pemphigoid

### Clinical Presentation

BP is the most common member of a family of autoimmune blistering diseases. BP primarily affects the elderly (age ≥ 60 years) and disease prevalence increases with age; worldwide estimates range from 12 to 66 new cases per million per year in the general population with rates increasing >12-fold in individuals over the age of 80 years (1–6). When adjusted for age, women exhibit a slightly higher risk of developing BP prior to 80 years of age, but the highest overall risk is observed in men aged ≥ 90 years (6). Disease prevalence is not impacted by race or ethnicity (1, 3, 6).

**Figure 1:**
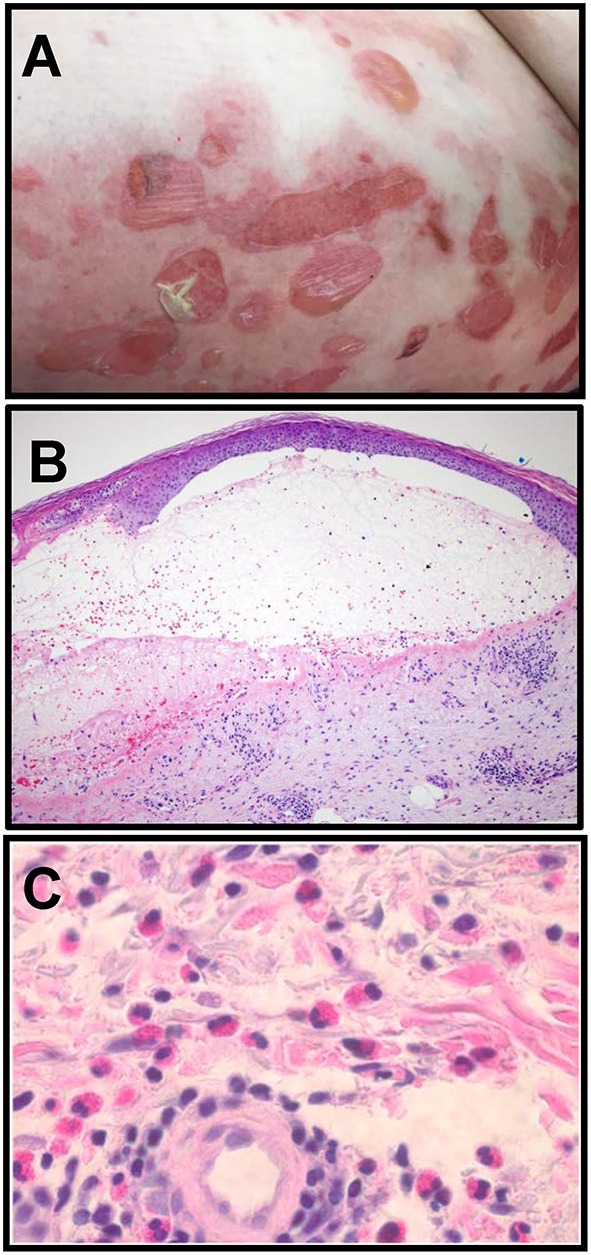
Clinical and histologic characteristics of Bullous pemphigoid. **(A)** Clinical presentation of BP with tense, fluid filled blisters occurring on areas of erythema and normal skin, frequently associated with urticarial plaques. **(B)** Blisters correspond histologically to a subepidermal separation at the basement membrane zone (BMZ) with eosinophils observed in the superficial dermis and the blister cavity. H and E, 100x. **(C)** Eosinophils in the deep perivascular infiltrate in a lesional biopsy from a BP patient. H and E, 400x (Images in **B**, **C** are courtesy of Dr. Brian L. Swick, University of Iowa).

The onset of classical BP is often preceded by a period of pruritis, followed by development of urticarial or eczematous lesions and the formation of tense, fluid-filled blisters on areas of erythema and normal skin (Figure 1A). Blisters correspond histologically with a subepidermal separation (Figure 1B) through the lamina lucida of the basement membrane zone (BMZ) (7). An inflammatory infiltrate comprised primarily of eosinophils, accompanied by lymphocytes, mast cells and neutrophils is observed (Figures 1B,C) (4, 7, 8). Immunologic criteria for BP include linear deposition of antibodies and/or complement (C3) at the epidermal BMZ and confirmation of circulating cutaneous autoantibodies via indirect immunofluorescence (IF) or ELISA (7, 9, 10).

The initial presentation of BP is heterogeneous; lesions can be localized to discrete areas or distributed widely over the body. There is no generally accepted classification of disease severity, although % affected body surface area is considered representative of mild (<10%), moderate (10–30%) and severe (>30%) disease, and can be used to inform initial treatment regimen (4). In an effort to standardize measurements of disease extent and facilitate comparison of therapeutic outcomes in multi-center studies, an international panel of experts developed the BP disease Area Index (BPDAI) score (11) which considers the number, size and anatomic location of lesions observed by the physician, as well as the duration and severity of itching as described by the patient. BPDAI scores range from 0 to 360 for BPDAI total activity (maximum 240 for total skin activity and 120 for mucosal activity), and a separate subjective measure known as BPDAI-pruritus. Standard treatments for BP include topical and systemic steroids, often in combination with adjuvant immunosuppressive or immunomodulatory therapy [reviewed in (4)]. BP often follows a chronic course, with complete remission (off therapy) achieved in months to years (4, 11).

### BP Autoantibody Subclass and Specificity

The cellular targets of BP autoantibodies are two hemidesmosomal proteins, BP180 (Type XVII Collagen) and BP230, involved in stable attachment of basal keratinocytes to the underlying matrix (12, 13). BP180 is a transmembrane protein that interacts with dermal matrix components, including integrin α6 and laminin-332, and BP230 acts as an intracellular linker of the hemidesmosomal plaque to keratin intermediate filaments (14). Expression of BP180 is restricted to stratified, pseudostratified and transitional epithelia (15), whereas tissue-specific isoforms of BP230 (also called Dystonin) are expressed throughout the body, including skeletal muscle and brain (16).

The pathogenicity of BP180-specific IgG (BP180 IgG) antibodies has been established experimentally, while the role of the BP230-specific IgG remains controversial (17). Accordingly, clinical disease activity often correlates with serum levels of antibodies targeting BP180, but not BP230 (18–20). It has been proposed that the development of BP230-specific antibodies may be secondary to the tissue destruction mediated by BP180 IgG (21). Historically, studies exploring disease pathogenesis have focused on IgG, although IgE- and IgA-class autoantibodies have also been described in BP (22).

The pathogenicity of the BP180 IgG can be attributed to two basic mechanisms: mechanical disruption of keratinocyte adhesion and immune-mediated events. Treatment of cultured keratinocytes or skin organ cultures with BP180 IgG results in internalization of BP180 from the cell surface and decreased keratinocyte adhesion (23–26). A corresponding decrease in both BP180 expression and hemidesmosomal localization to the cell surface is also observed. At the same time, IgG autoantibody deposition triggers complement activation and recruitment and activation of immune cells, resulting in release of destructive proteases and ongoing inflammation (27–29). While IgG-based models were critical for understanding the fundamental pathomechanisms of BP, they failed to recapitulate the itching, erythema and eosinophilia observed in human disease (30–32). Thus, the pathogenic contribution of IgE in BP was considered based on the early urticarial phase of BP and the established role of IgE in Type I hypersensitivity responses.

## IgE Autoantibodies in BP

### Incidence and Specificity

Elevated levels of circulating IgE and linear deposition of IgE at the BMZ of biopsied skin were first reported nearly 50 years ago (32, 33); however, the role of IgE in disease pathogenesis remained largely unexplored for several decades. It wasn't until 2007 that detailed epitope mapping studies revealed that the IgE autoantibodies primarily target the same non-collagenous 16A (NC16A) region of BP180 that is recognized by BP IgG (30, 31). Subsequently, the number of published manuscripts examining IgE in BP has increased steadily (34). Together, these reports have established that elevated circulating IgE is observed in most (70–85%) BP patients while the reported incidence of BP180 specific IgE (BP IgE) varies widely (22–100%) (34–38). This variation results from differences in autoantibody detection methods, lack of optimization for detection of IgE and heterogeneity of the patient population (34, 35, 39).

In most studies, total circulating IgE is measured by clinical reference labs using standardized procedures and reagents; however, commercial reagents have not been developed to measure BP180 IgE. To address this, individual labs have developed immunoblot or ELISA protocols utilizing their own recombinant protein antigens (30, 40–44) or employ the antigen-coated plates from a commercial BP180 IgG ELISA paired with an IgE-specific secondary antibody (37, 45–47). Additionally, Pomponi et al. (48) developed a microarray system that has potential for simultaneous assessment of both IgE and IgG specific for the NC16A domain of BP180. Despite the heterogeneity of the assays employed, studies show that most (61–77%) BP patients have both IgG and IgE specific for BP180 in their sera (48–50). Additionally, there is increasing evidence that BP230-specific IgE antibodies are prevalent in BP (44, 46, 50–52) and IgE antibodies specific for epitopes within the intracellular domain of BP180 have also been reported (30), although their clinical significance is not known.

### Clinical Findings Associated With IgE Autoantibodies

#### Disease Severity or Phenotype

In an effort to better understand the clinical relevance of IgE in BP, studies have examined whether elevated IgE antibody levels are associated with a particular disease phenotype (Table 1). Most studies report that circulating total IgE levels are directly correlated with disease severity in all or a subset of patients with high IgE and that IgE levels decline as disease resolves (33, 45, 47, 49, 53). Not surprisingly, IgE deposition at the BMZ is observed more often in patients with high circulating IgE (59, 63), which likely reflects the interference of excess amounts of IgG and difficulty of detecting ng/ml IgE concentrations. Furthermore, detection of bound IgE is complicated by the cross-binding of anti-IgE antibodies to IgG (43, 50). Given the abundance of specific IgG at the BMZ, it is difficult to know what proportion of IgE reactivity is specific without stringent validation of antibody specificity. This can be achieved by testing the reactivity of secondary reagents to patient serum antibodies after removal of IgG via immunoadsorption and/or 2-step affinity purification (removal of IgG followed by enrichment of IgE) (43, 50, 64). Unfortunately, this type of secondary antibody validation is often not done or not reported. Inconsistencies in the sensitivity and specificity of these assays likely contribute to the variability in the reported rates (18–65%) of IgE antibody deposition *in vivo* (24, 33, 53, 54, 59, 63, 65). This variability precludes a reliable association of *in vivo* IgE deposition at the BMZ with a single disease phenotype.

**Table 1:** Association of serum IgE antibody levels with severity or phenotype of Bullous pemphigoid and eosinophilia as reported in primary literature^1^^,^^2^.

	**Disease severity**		**Disease phenotype**			**Eosinophilia**	
	**Positive association**	**No association**	**Urticarial**	**Nodular**	**None**	**Peripheral or lesional**	**None**
Total IgE	(33, 47, 49)	(45, 53)	(37)	(45)		(49)	(54)
BP180 IgE ELISA	(37, 41, 47, 49, 55–57)	(46, 58–60)	(57, 61)	(45, 50)	(37)	(49)	(50, 57, 60)
BP230 IgE ELISA	(50, 62)	(46, 50, 56, 59) (negative association)		(50)		(46)	(50)

1 *Including case series, case control, cohort, and retrospective studies*.

2 *Total IgE was measured using a commercially available ELISA or by chemiluminescence at institutional reference labs. BP180- and BP230-IgE was measured using lab-specific protocols using either recombinant protein antigens made in-house or antigen-coated plates from a commercial BP180 IgG ELISA paired with an IgE-specific secondary antibody*.

**Figure 2:**
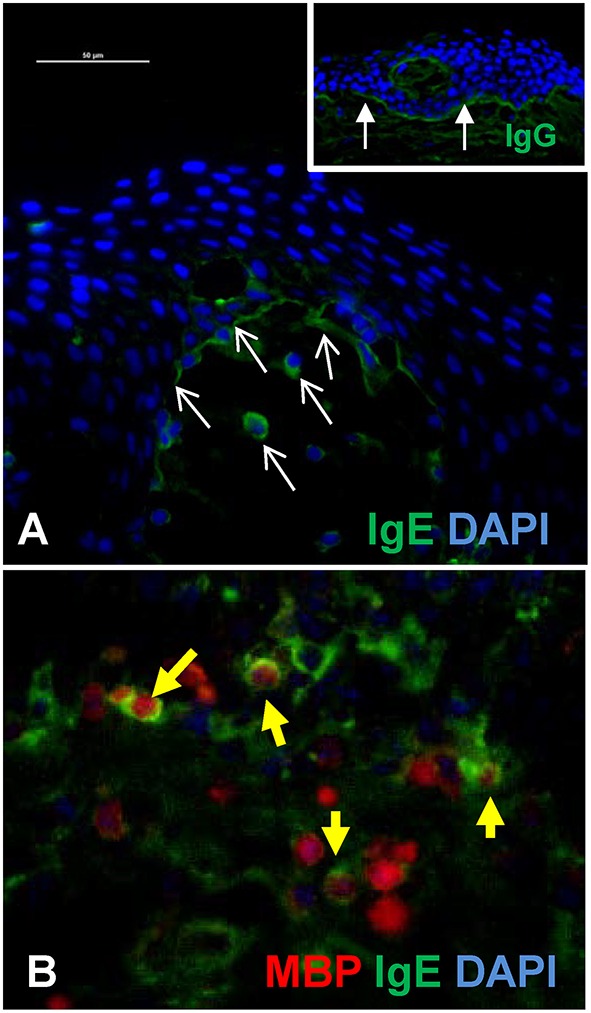
IgE antibodies localize to the BMZ and eosinophils in BP lesions. **(A)** Indirect immunofluorescent staining of autoantibody deposition in BP lesions reveals IgE deposition at the BMZ and on infiltrating cells in the superficial dermis (white arrows = IgE). Inset image shows robust IgG deposition (filled arrows) in an adjacent section from the same patient. Scale bar = 50 μM. **(B)** Immunofluorescent staining of lesional skin from a BP patient reveals eosinophils (major basic protein, red) and IgE (green) in the deep cellular infiltrate. IgE coated eosinophils are indicated by the yellow filled arrows. DAPI nuclear stain (blue). The specificity of the secondary antibodies (anti-human IgE, A80-109A; anti-human IgG, A80-148P) was tested by dot blotting which confirmed little cross-reactivity to mg concentrations of each antibody isotype and no cross reactivity to <0.5 mg to the differing isotype, while their specific reactivity to all concentrations (0.1–1 mg) was robust.

Figure 2A is an example of indirect immunofluorescent staining for the detection of IgE and IgG at the BMZ of a lesional biopsy of a BP patient. As previously described, bright linear staining is observed with anti-IgG (inset), while the anti-IgE staining at the BMZ is less robust and IgE-coated cells are seen in the superficial dermis (Figures 2A,B) (24, 42, 59, 66). In this experiment, the specificity of the secondary antibodies was tested against serial dilutions of human IgG or IgE by immunoblot.

Similar to total IgE, most studies examining BP180-specific IgE levels in BP find a positive association with disease severity, clinical course or disease outcome [reviewed in (34)]. In some cases (Table 1), circulating concentrations of BP180-specific IgE correlated with the number or area of skin lesions or BPDAI scores (37, 41, 47, 49, 55, 56), while others find no association (46, 58–60). The link between specific IgE and disease phenotype is inconsistent; some studies correlate specific IgE with prominent urticaria (57, 61), others find an association with a nodular BP phenotype (45, 50), and some show no association (37). Finally, others suggest that high IgE autoantibody levels serve as a marker of patients who require a longer and more aggressive treatment for remission (55, 56).

A handful of studies have measured BP230-specific IgE in patient serum using a commercially available ELISA plate paired with an anti-IgE detection antibody (Table 1). These reports suggest that BP230 IgE serves as an index of either overall disease activity (62), or lesional eosinophilia (46), while others find no association (50, 56, 59). One report (46) found that BP230 IgE levels were inversely related to overall disease severity. Somewhat surprisingly, one study found that serum levels of BP230 IgE associated with the nodular, not erythematous, disease phenotype (50). Although the impact of IgE autoantibodies, targeting either BP180 or BP230, on disease phenotype remains unclear, implementation of the BPDAI scoring system will provide increased clarity as will standardization of sensitive and specific assays optimized for measuring antigen-specific IgE due to competition with much higher concentrations of specific IgG (37, 43, 50, 64).

#### IgE Receptors

IgE production and its downstream effects are regulated by a complex network of cell-bound and soluble receptors. The cell-bound receptors, FcεRI and CD23/FcεRII are involved in regulation of IgE production and activation of innate immune cells, including APCs, macrophages and granulocytes (67, 68). The high affinity receptor, FcεRI, is primarily responsible of antigen-specific degranulation of mast cells and basophils, whereas CD23 is widely expressed on B cells, where it plays a key role in antigen focusing and stimulation of a Th2 response. Both receptors are positively regulated by circulating IgE thereby amplifying its systemic effects (67). Thus, it is not surprising that increased cellular expression of IgE receptors is observed in BP. One study noted increased expression of both CD23 and FcεRI on circulating eosinophils and basophils from BP patients, but levels were variable and did not correlate with circulating IgE (49). The implications of this finding will be discussed in the *Evidence for interaction of IgE and eosinophils in BP* section below. Inaoki et al. (69) found that peripheral B cells from BP patients exhibited increased expression of CD23, which correlated with both circulating IgE levels and disease severity. These observations suggest that modulation of FcεRI and CD23 plays a role in the generation and/or maintenance of the IgE autoantibody response in BP.

In addition to the cellular receptors, soluble (s) IgE receptors, sCD23, sFcεRI, and Galectin-3, are key components of the IgE network [reviewed in (68)]. The best studied soluble receptor, sCD23, is thought to be a positive regulator of IgE production, whereas the biologic roles of sFcεRI or Galectin-3 are not well-defined. Circulating levels of sCD23 have been explored as a potential biomarker of disease activity in number of IgE mediated diseases (68). Similarly, sCD23 levels are elevated in the serum and blister fluid from BP patients and are associated with circulating IgE levels and increased disease severity (69–73). The modulation of sCD23 levels in BP suggests that specific targeting of this receptor may prove effective in reducing IgE antibody levels (74). To date, no studies have examined sFcεRI or Galectin-3 in BP.

#### Cytokine Profiles

While IgE autoantibodies are occasionally reported in other autoimmune blistering diseases, BP is unique in that most BP patients develop an IgE autoantibody response. Although it is not known what triggers an IgE response in BP, it is known that IgE antibody production is driven by Th2 cytokines. Specifically, B-cell class switching to IgE is dependent on IL-4, IL-13, and CD40 ligation, while IL-5 enhances antibody production (75, 76). In diseases associated with IgE, circulating antibody concentrations often correlate with levels of Th2 cytokines (77, 78). Accordingly, systemic expansion of the Th2 population and increased levels of Th2 cytokines and chemokines are detected in serum and blister fluid from BP patients, along with several other drivers of autoimmunity and inflammation (79–84). Of particular relevance, elevated levels of IL-4 and IL-5 undoubtedly facilitate the IgE autoantibody response and upregulate cellular and soluble CD23 expression (68, 75, 76). Notably, no studies to date have defined clear relationships between expression of specific cytokines and IgE antibody levels in BP patients.

### Demonstration of IgE's Pathogenicity in BP

#### Experimental Evidence

The pathogenic mechanisms of BP IgE have been explored *in vitro* using cultured keratinocytes or skin organ cultures treated with BP IgE or monoclonal IgE specific for NC16A (24, 85). In both systems, *in vitro* treatment with IgE autoantibodies resulted in internalization of BP180 from surface of basal keratinocytes and increased secretion of IL-6 and IL-8, cytokines shown to play a key role in IgG-based models of murine BP. A decline in keratinocyte adhesion and hemidesmosomal density was also observed in IgE treated cultures (24, 85). These observations are comparable to studies utilizing BP180-specific IgG, suggesting that the antibody mediated disruption of mechanical adhesion is not isotype specific.

The *in vivo* pathogenicity of BP IgE was initially explored in two mouse models utilizing passive antibody transfer. In the first, IgE purified from patient serum was injected into human skin that had been grafted onto nude mice (86). Injection of physiologic levels (6–47 ng/graft) of IgE recapitulated clinical disease in the grafted skin with linear IgE deposition at the BMZ, development of erythematous plaques, eosinophilic infiltration, and a histologic subepidermal split. Similar observations were made in the second model, which utilized intradermal injection of an IgE hybridoma specific for the BP180 ectodomain into SCID mice (87). These studies were unique because they were the first passive transfer experiments to fully replicate clinical disease, with eosinophil infiltration and frank blistering, and they were the first to demonstrate the pathogenicity of IgE autoantibodies *in vivo*.

#### Clinical Evidence

Based on the elevated IgE and early urticarial phase of disease and the experimental evidence demonstrating the pathogenicity of IgE autoantibodies in mice, anti-IgE therapy was evaluated for clinical BP. Omalizumab (OMZ) is a humanized monoclonal antibody that binds free IgE, thereby blocking subsequent interaction with its receptors, and is approved for treatment of severe asthma and chronic urticaria in both the US and Europe (88, 89). In 2009, the successful treatment of a recalcitrant BP patient with OMZ as a monotherapy provided the first definitive evidence of a pathologic role for IgE autoantibodies in human autoimmunity (90). This patient was selected due to failure of traditional therapies, elevated circulating total IgE (222 IU, normal < 100) and peripheral eosinophilia. One week after the first dose, the intact blister count decreased by 44% and after 16 weeks, blistering resolved and eosinophil counts declined to near normal [3,427 to 460/mm^3^ (normal < 400)], while IgG autoantibody levels remained high. Based on its initial success, OMZ has been used as a monotherapy or steroid sparing agent in 22 patients [reviewed in (91)]. A more detailed discussion of OMZ treatment of BP is found in the *Therapies targeting IgE and Eosinophilia* in BP section below.

## Eosinophils

### Introduction

Eosinophils are best known as end-stage effector cells associated with allergy and parasitic infection. However, eosinophils are increasingly recognized for their roles in immunomodulation, tissue remodeling and repair, and autoimmunity (92, 93). Under homeostatic conditions, eosinophils represent 1–5% of circulating leukocytes and are observed infrequently in the skin, but their numbers are often elevated in allergic, neoplastic and immunologic skin disorders (94).

Eosinophil expansion, trafficking and survival are dependent on IL-3, IL-5, and GM-CSF (95–98). Specifically, IL-5 stimulation of bone marrow precursors leads to increased numbers of circulating eosinophils that then enter tissues in response to Th2-dependent (IL-4 and IL-13) expression of chemokines, including eotaxins (CCL11, CCL24, CCL26), CCL5 (RANTES), endothelial cell vascular cell adhesion molecule 1 (VCAM-1), PAF, and complement (C5a) (41, 99). Once in the tissues, most eosinophils do not recirculate and have a short life span of 2–5 days that can be prolonged by several cytokines, including IL-3, IL-5, IL-33, GM-CSF, and IFN-ɤ (99). Mature eosinophils express a variety of receptors, including those for immunoglobulins (IgG, IgA, IgE) and complement (CR1, CR3, CD88), in addition to a number of cytokines and chemokines, including those mentioned above (99). Under certain conditions, tissue eosinophils play an additional role in antigen presentation, which is facilitated by upregulation of MHC II and co-stimulatory molecules CD80/CD86 (97, 100, 101).

Eosinophils exert their anti-pathogen and immunoregulatory functions through the regulated release of pre-formed granules and newly synthesized proteins that include over 35 cytokines, chemokines and growth factors (95, 99, 102–105). Through selective release of these mediators, eosinophils influence immunity and tissue homeostasis (93). Eosinophil specific granules contain four unique proteins: major basic protein (MBP), eosinophil cationic protein (ECP), eosinophil peroxidase (EPO), and eosinophil-derived neurotoxin (EDN) (106). Upon activation, these toxic proteins aid in the elimination of microbes, parasites and tumor cells. IL-5 and GM-CSF are considered the most effective and specific signals for inducing eosinophil activation, enabling release of specific proteins in response to environmental signals (93, 107). In addition, exposure to GM-CSF, IL-5, IFN-ɤ, eotaxin, and TSLP can facilitate the extrusion of mitochondrial DNA traps, known as eosinophil extracellular traps (EETs), which provide additional microbicidal functions by ensnaring pathogens and facilitating contact with granules or toxic granule proteins (108–110).

### Eosinophils in BP

#### Eosinophilia

Although peripheral and lesional eosinophilia is a prominent feature of BP (54, 111, 112), the association of a disease phenotype with the degree of eosinophilia has only recently been evaluated. Most of these studies show that both circulating and lesional eosinophil numbers are closely associated with the extent and severity of disease in untreated patients and also those undergoing standard immunosuppressive therapy (45, 49, 113, 114). A recent study of 65 well-defined BP patients found a correlation between circulating eosinophil numbers and the extent of disease activity (number of blisters and erosions), but not the extent of urticaria or erythema (36). This observation is somewhat surprising based on the established role of eosinophils in allergic urticaria; however, it is possible that an enumeration of lesional eosinophil numbers would be more relevant for this analysis.

In BP, eosinophilia has been associated with increased levels of factors associated with eosinophil expansion, survival and chemotaxis. Produced in the skin, many of these factors play a dual role, fostering both chemotaxis and survival of migrating cells (115). In particular, eotaxin levels are correlated with lesional eosinophilia in BP (81, 82, 112, 116–118). To a lesser extent, IL-5 levels in the serum and blister fluid have also been associated with degree of eosinophilia in BP (119). Eosinophil accumulation in lesional skin is further facilitated by their upregulation of corresponding cytokine and chemokine receptors (118).

#### Eosinophil Activation

In many diseases featuring eosinophilia, release of cytolytic granule proteins is used as an index of eosinophil activation (106, 120). In BP, increased activation of eosinophils is demonstrated by elevated levels of ECP, MBP, and EPO, in the skin, blister fluid, and to a lesser extent, the circulation (119, 121–123). Serum levels of ECP and EDN have been correlated with disease activity and, accordingly, these levels are decreased in patients receiving immunosuppressive therapy (119, 121). One study found that an initial reduction in serum ECP concentration was associated with an increased likelihood of remission in the first year of treatment (124). Additionally, the fragility of the BMZ is further enhanced via upregulated eosinophil expression of matrix metalloproteinase 9 (MMP-9), and the localized release of EETs that enhance tissue destruction (125–128).

Within BP lesions, eosinophils exhibit increased expression of activation markers, and the presence of degranulated cells alongside extracellular granules confirms *in situ* degranulation (111, 121, 129–133). Eosinophil degranulation is most prominent in early erythematous and urticarial lesions, precedes blister formation, and is not observed in uninvolved skin (111). Studies using a human cryosection model of BP suggest that eosinophil localization to the BMZ is dependent on complement fixation, but BP autoantibodies and complement are not sufficient to induce subepidermal separation (123, 128). Instead, priming of eosinophils with IL-5 was essential for their release of destructive mediators that were essential for separation at the BMZ (128). The clinical association between the number of BP lesions and IL-5 levels in the blister fluid increases the likelihood that eosinophils play an integral role in loss of epidermal adhesion (119).

### Evidence for Interaction of IgE and Eosinophils in BP

Histologic studies suggest an indirect route of IgE-mediated eosinophil activation, via tissue mast cells, since mast cell degranulation precedes eosinophil infiltration into new lesions (112, 131, 132, 134). Indeed, mast cells constitutively express high levels of IgE receptors and are known to release mediators of eosinophil migration, such as IL-5 (135). The likelihood of a direct contribution of mast cells to lesion development is supported by the detection of dermal mast cells coated with both IgE and BP180 peptides (42, 66), and *in vitro* experiments demonstrating that peripheral basophils from BP patients degranulate upon exposure to BP180 peptides (42). Within the skin, naturally shed fragments of BP180 would facilitate autoantibody-specific degranulation. In response to these signals provided by mast cells, eosinophil accumulation and degranulation in the skin will then trigger production of additional chemotactic and inflammatory factors by keratinocytes, resulting in a positive feedback loop of eosinophil recruitment and activation (136).

While mast cells are undoubtedly a main mechanism of IgE-mediated eosinophil activation in BP, the co-localization of IgE antibodies and BP180 fragments in BP lesions indicates that direct interaction of IgE and eosinophils also occurs (42, 49, 66). However, studies aimed at defining the exact nature of their interaction have been hampered by numerous technical difficulties, including the relative rarity of eosinophils, lack of a specific cell lineage marker to facilitate their identification and purification, and their propensity for non-specific degranulation. Additionally, *in vivo* studies are complicated by differences in cellular distribution of IgE receptors across species and the inability of human IgE to bind murine IgE receptors. Despite these challenges, a handful of studies provide additional support for direct modulation of lesional eosinophils by BP IgE.

Initially, a route of direct interaction between IgE and eosinophils in BP was not well-received since eosinophils from healthy donors do not express FcεRI. However, eosinophil expression of FcεRI has been reported in diseases characterized by high IgE and eosinophilia (137–139). Similarly, mRNA and/or cell surface-bound FcεRI are observed in circulating and lesional eosinophils from BP patients, although these studies did not prove receptor functionality (49, 55, 140). However, antibody binding experiments conducted on perilesional skin sections suggest that IgE binding is dependent on FcεRI, but not CD23 (66). Finally, comparison of FcεRI receptor chains expressed by circulating and lesional eosinophils suggests that while both populations express the trimeric (αγ_2_) form, lesional eosinophils may also express tetrameric (α*βγ*_2_) form that is known to mediate degranulation (49, 67).

The first *in vivo* evidence suggesting a link between IgE and eosinophils was provided by the previously discussed IgE-based passive transfer mouse models of BP (86, 87). Notably, administration of IgE autoantibodies resulted in eosinophilia, erythema, and pruritus. These symptoms are often observed clinically but were absent in IgG-based mouse models. In further parallel to clinical disease, IgE-coated mast cells and degranulated mast cells were also observed in IgE treated mice. Thus, these initial studies did not determine whether the interaction of IgE and eosinophils was direct or indirect (through mast cells). However, convincing evidence of a direct interaction between IgE and eosinophils in BP was provided via treatment of double humanized mice, expressing human NC16A and human FcεRI, with NC16A-specific IgE (141). In this model, disease severity was IgE dose dependent and was directly related to the degree of cutaneous eosinophilia. Additionally, eosinophils were required for IgE-mediated blister formation. These observations are mirrored in OMZ-treated BP patients, where disease activity is closely paralleled by peripheral eosinophil numbers, rather than IgG autoantibody levels (90). Unfortunately, determination of serum levels of active vs. inactive IgE (OMZ bound) is not routine, so it is not known how functional IgE levels correlate with eosinophilia in these patients.

## Therapies Targeting Ige and Eosinophilia in BP

There are currently no approved drugs for treatment of BP, so current therapy relies on non-specific suppression of antibody production and inflammation with topical or systemic steroids and immunosuppressants. This results in significant morbidity and mortality in elderly BP patients (142). Thus, therapies targeting key aspects of disease pathogenesis are an area of intense interest for BP. Treatments that modulate IgE autoantibody levels or inhibit the downstream effects of IgE, as well as those targeting the Th2 axis or eosinophils are discussed below. Typically, these treatments are initially utilized on refractory patients who have failed standard immunosuppressive therapies, although recently some have been evaluated for their efficacy as a first-line treatment in clinical trials. Successful treatment is minimally defined as an absence of new lesions and a resolution of ~80% of existing lesions. In most of these studies, total and BP antigen-specific IgE are not reported, possibly due to the lack of standardized, commercially available assays. Development of standardized methods for the detection of IgE autoantibodies is necessary to fully understand the mechanisms responsible for therapeutic efficacy or failure in BP.

### Therapies Aimed at Reducing IgE Antibody Levels

#### Immunoadsorbtion

Immunoadsorption is used to non-specifically remove antibodies from the plasma of patients with severe disease and high autoantibody levels (143). It is thought that the sharp decline in circulating antibody levels leads to re-diffusion of tissue-bound antibodies, thereby and alleviating their local effects in the skin (144). Treatment of BP with adjuvant immunoadsorption results in a durable decrease in disease severity and BP IgG (145, 146). Although IgE is usually not measured in BP patients treated with immunoadsorption, it is likely that both IgG and IgE are decreased, based on a report showing that pan-immunoadsorption effectively reduced serum IgE by >90% in patients with severe atopic dermatitis (147). Although not yet tested in BP, IgE-specific immunoadsorption has recently become available for clinical use (147–149). Due to the associated risks of infection immunoadsorption is typically used as a stop-gap measure to provide acute relief while immunosuppressive therapies are optimized (150).

#### B-Cell Depletion

Selective B cell depletion via targeting of CD20, a B cell lineage marker, has been utilized in BP to reduce circulating autoantibody levels and alleviate disease activity. Rituximab (or Rituxan) is a CD20-specific monoclonal antibody that eliminates circulating memory B cells and short-lived plasma blasts but leaves bone marrow plasma cells intact due to their lack of CD20 expression. To date, reports of rituximab therapy for BP consist of largely of retrospective analysis of refractory patients treated with a variety of dosing regimens. Overall, patients showed dramatic improvement (facilitating tapering of prednisone) but IgE antibody levels were not reported (151–153). Thus, it is impossible to know whether patients with high IgE autoantibody levels respond similarly to rituximab or if recurrence is associated with persistence of IgE autoantibodies. A single report found that rituximab treatment leads to a sharp decrease in IgG, while IgE autoantibody levels are slower to respond (154). This observation suggests CD20- plasma cells contribute to IgE autoantibody levels, whereas IgG antibodies are produced by CD20+ short lived plasma blasts (1).

A recent systematic review found 85% of BP patients treated with rituximab exhibit a complete response (no new lesions or pruritic symptoms and healing of at least 80% of lesions) with or without other therapies (11). Recurrence rates and reported adverse events were each observed at a rate of 25% (11). Another retrospective analysis examined efficacy of rituximab treatment patients with pemphigoid diseases found that 5/8 BP patients achieved initial disease control with rituximab, and 5/8 achieved partial remission; however, 5 patients suffered a relapse and there was one death, possibly related to treatment (153). The results of an open-label, prospective, phase 3 clinical trial evaluating the efficacy and safety of a single cycle of rituximab (two infusions of 1,000 mg, 15 days apart) for the treatment of BP (NCT00525616) are not yet available.

#### IgE Blockade

The downstream effects of IgE antibody interaction with immune cells have been targeted using omalizumab (Xolair), a humanized monoclonal antibody that binds the Fc portion IgE thereby inhibiting high affinity receptor interaction (155, 156). Dosing is determined as in asthma, based on total serum IgE levels and patient body weight, or as used in chronic urticaria, 150 or 300 mg every 2 weeks (89, 155). OMZ treatment leads to a reduction in B cell production of IgE, decreased activation and degranulation of mast cells and eosinophils, and a dramatic decline in peripheral eosinophil numbers (155–158). To date, OMZ has been used as a monotherapy or steroid sparing agent in 22 patients described in several case reports and series [reviewed in (91)]. Most of these patients had high IgE levels (73%) and eosinophilia (77%) and nearly all (93%) had been unsuccessfully treated with systemic corticosteroids. Although initial dosage and duration of treatment varied considerably among patients, 85% of patients undergoing OMZ therapy exhibited a complete response [no new lesions or pruritic symptoms and healing of at least 80% of lesions (11)] on or off other therapies. In addition, most patients exhibited a dramatic decrease in eosinophil counts and decreased use of immunosuppressants. Importantly, several OMZ-treated patients exhibit dramatic clinical improvement despite persistently elevated IgG autoantibody levels (90, 159, 160). The drawbacks of OMZ therapy are that recurrence rates are high (84% within 3.4 ± 1.9 months), necessitating repeated cycles of OMZ, and corticosteroids or immune suppressants are often needed to control disease. Furthermore, 20% of treated patients experienced adverse effects, such as thrombocytopenia, elevated liver enzymes, and myocardial infarction (two patients, resulting in one death) (91).

QGE031 (ligelizumab) is an anti-IgE antibody that binds IgE with higher affinity than OMZ. After promising results as a treatment for persistent hives (161), the efficacy and safety of QGE031 was examined in the only randomized, double blind, placebo-controlled study to date that evaluates the effects of directly targeting IgE in BP (NCT01688882). Unfortunately, the trial was halted after the first part did not achieve the predefined criteria of efficacy (>50% better than placebo). Based on the relative success of OMZ therapy for BP (discussed above), it is surprising that QGE031 wasn't found to be beneficial. There are some differences in study design and treatment approach that could contribute to this result. First, the criteria for enrollment in the QGE031trial were BP patients, aged 20–80, with disease refractory to oral steroid treatment and total IgE levels up to 5,000 IU/ml; however, patients were not selected based on elevated IgE levels (or eosinophilia). Since OMZ-treated patients are typically selected based on elevated IgE and eosinophilia, its efficacy has not been examined in patients who do not have high IgE. Secondly, the efficacy of QGE03 (240 mg), given subcutaneously every 2 weeks aimed to reduce disease activity by >50% after 12 weeks of treatment. In contrast, off-label OMZ therapy for BP is typically administered every 2 weeks for 16 weeks (1 cycle), patients often require multiple cycles (at the discretion of an unblinded provider), and there is no standardized benchmark of success. Thus, OMZ treatment regimen is often tailored to each patient, depending on the initial response (91). It is possible that the shorter duration of treatment and stringent requirement for >50% improvement influenced the outcome of the QGE031 trial. Although a detailed description of the QGE031 trial has not been published, these findings would be of high importance to the field, despite a negative result. Finally, the therapeutic success of OMZ has not been tested in a randomized, double blind, placebo-controlled trial, which is essential for the elimination of bias.

#### Intravenous Immunoglobulin

Intravenous immunoglobulin (IvIg) is a highly effective therapy for BP that is typically used as an adjuvant in combination with an immunosuppressive agent (144). The clinical efficacy of IvIg has been demonstrated; however, their mechanism of action is not well-understood (162–164). In mouse models of BP, IvIg treatment ameliorated skin fragility, decreased serum levels of inflammatory cytokines and chemokines and reduced circulating IgG autoantibody levels. *In vitro* studies suggest that anti-idiotypic antibodies present in IvIg might be responsible for its therapeutic effects in BP (165). In these studies, the addition of IvIg to keratinocyte cultures restored Collagen XVII expression and increased adhesion that had been reduced by BP treatment with BP IgG. Depletion of the anti-idiotypic antibodies ameliorated the beneficial effects of IvIg. The effects of IvIg on IgE autoantibodies in BP has not been explored, although it is established that IvIg also suppresses IgE production *in vitro* (166, 167) and *in vivo* (168) and anti-idiotypic antibodies targeting IgE have been described in allergy (169, 170).

A recent randomized, placebo controlled, double blind trial was conducted to investigate the therapeutic effects of IvIg on BP patients who showed no symptomatic improvement with ≥0.4 mg/kg/day prednisolone (NCT01408550). Adjuvant IvIg resulted in a significant decline in disease activity and a reduction in BP180-specific IgG, but IgE levels were not reported (164). While this study demonstrated a clear benefit of IvIg for treatment of BP, both IgE autoantibody levels and anti-idiotypic antibodies should be evaluated in future studies to better understand its mechanism of action.

### Targeting the Th2 Axis

Pharmacologics targeting the Th2 axis have been developed for use in asthma and allergy to block cytokines or chemokines critical for disease pathogenesis. These drugs are predominantly human monoclonal antibodies that specifically block receptor-ligand interaction but are also effective at reducing the overall Th2 response due to interruption of positive feedback loops (160). Due to the predominance of Th2-phenotype, including IgE autoantibodies and eosinophilia, many of these same medications have been used off-label to treat refractory BP.

Bertilimumab (anti-eotaxin-1) treatment of BP was examined in an open-label Phase II study (NCT02226146) in 9 patients with moderate to severe BP. Although IgE antibody levels have not been reported, preliminary analysis found an 81% decrease in BPDAI scores and a significant steroid sparing effect with bertilimumab therapy. Based on these effects, bertilimumab has been granted fast track designation as an orphan drug for the treatment of BP (171).

Used in moderate to severe atopic dermatitis, dupilumab (anti-IL4 receptor α) inhibits both IL-4 and IL-13 through their shared usage of the IL-4 receptor α chain. There is a single case report describing successful dupilumab therapy for a case of treatment-refractory BP (172). After 3 months of dupilumab, the patient reported decreased itching, IgG autoantibodies were not detectable, and lesions had resolved. The effect of dupilumab therapy on circulating IgE antibody levels or eosinophils was not reported. There are currently no clinical trials examining efficacy of dupilumab in BP.

Lastly, the effect of mepolizumab, an IL-5 inhibitor, was evaluated as an add on therapy (vs. placebo) to oral corticosteroids in patients an acute flare of BP [NCT01705795 (173)]. Patients treated with mepolizumab did exhibit any therapeutic benefits, such as decreased time to relapse or increased disease control, over the placebo group; however, significantly lower peripheral blood eosinophil levels were noted, and skin infiltrating eosinophils were also reduced. IgE antibody levels were not reported. Although a reduction in disease activity was not observed, the authors argue for continued exploration of therapeutic strategies targeting eosinophils in BP, such as antibodies targeting IL-5 receptor alpha subunit, since they will mediate antibody-dependent cell-mediated cytotoxicity of both eosinophils and basophils (174).

## Conclusions

While IgE autoantibodies and eosinophilia are established features of BP, their precise contribution to disease pathogenesis remains unclear. Experiments aimed at understanding the interaction between IgE and eosinophils are complicated by a web of shared mediators and feedback loops that cross regulate multiple components of Th2 immunity. However, use of transgenic and knockout mouse models of BP will improve experimental clarity. Additionally, the development of standardized assays for sensitive and specific measurement of BP180 IgE is needed to improve consistency of clinical studies. Going forward, consistent identification of patient characteristics, including total and specific IgE levels or degree of eosinophilia, will facilitate selection of targeted therapies to optimize patient outcomes.

## Author Contributions

KM: substantial contributions to the conception and design of the work and acquisition, analysis, or interpretation of data for the work, preparation and finalization of manuscript. TC: acquisition of data for the work. JF: critical evaluation of written content. KM, TC, and JF: contributed to manuscript revision, read, and approved the final version to be published.

### Conflict of Interest

The authors declare that the research was conducted in the absence of any commercial or financial relationships that could be construed as a potential conflict of interest.
